# Spatiotemporal Distribution of Tuberculosis and COVID-19 During the COVID-19 Pandemic in Libya

**DOI:** 10.1017/dmp.2020.458

**Published:** 2020-11-19

**Authors:** Mohamed A. Daw, Faraj A. Zgheel, Abdallah El-Bouzedi, Mohamed O. Ahmed

**Affiliations:** 1 Department of Medical Microbiology and Immunology, Faculty of Medicine, University of Tripoli, Tripoli, Libya; 2 Department of Pharmacology, Biotechnology Research Center, Tripoli, Libya; 3 Department of Laboratory Medicine, Faculty of Biotechnology, University of Tripoli, Tripoli, Libya; 4 Department of Microbiology and Parasitology, Faculty of Veterinary Medicine, University of Tripoli, Tripoli, Libya

**Keywords:** tuberculosis, COVID-19, temporal ananlysis, geographic analysis

The coronavirus disease (COVID-19) pandemic has posed serious health and economic threats, particularly in developing countries. The presentation of the disease is highly variable and could be easily confused with other respiratory tract infections.^1^ In Africa, tuberculosis (TB) is one of the top causes of mortality and has a presentation conspicuously similar to the current severe acute respiratory syndrome coronavirus 2 (SARS-CoV-2) infection. During the past coronavirus epidemics like SARS and Middle East respiratory syndrome-related coronavirus (MERS-CoV), co-infections with TB had posed a major threat to the spread of the disease. Hence, the association between COVID-19 and TB cannot be ruled out, and more evidence should be gathered to increase our understanding of the dynamics of both diseases during the spread of the pandemic.^2^ Therefore, it is important to understand the distribution and aggregation degree of TB and COVID-19 and to follow up the spatial trends of both of them during the pandemic period. In this study, we aimed to analyze the spatiotemporal variation and the trends of TB and COVID-19 during the pandemic at the national and regional levels. This will provide more information and thus help implement proper strategies to combat the burden of the pandemic.

To investigate the epidemiological association between TB and COVID-19 infections, we traced all the notified TB cases and the cumulative number of COVID-19 cases during the emergence of the COVID-19 pandemic in Libya. The time and spatial distribution of COVID-19 and TB cases were compared, and the correlation between both of them was determined. The spatial autocorrelation analysis was conducted by using open GeoDa software v1.2.0 (GeoDa Center for Geospatial Analysis and Computation, Arizona State University, Tempe, AZ) and creating thematic mapping, as previously described.^3-5^

**Figure 1.**
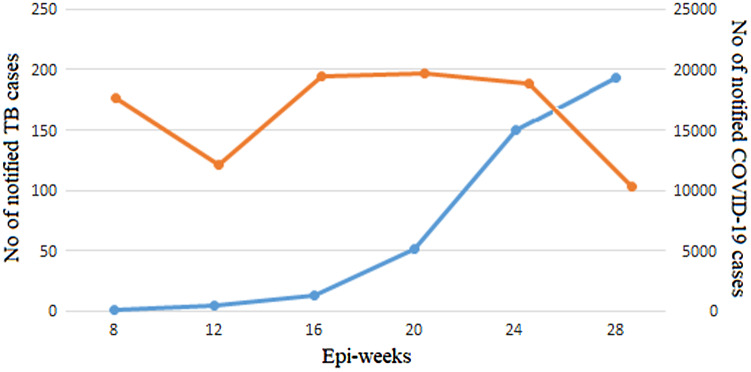
The number of notified cases of TB (yellow) and COVID-19 (blue) during 28 epi-weeks of COVID-19 in Libya.

The number of notified TB and the cumulative number of COVID-19 cases were reported during the first 28 epi-weeks of the pandemic spread, as shown in Figure 1. Starting from March 23, when the first case was identified in Libya, till October 5, 2020^6,7^ – 41 686 COVID-19 cases were documented throughout the country during those first 28 epi-weeks. Only 75 cases were reported in the first 8 weeks, followed by 466 and 1247 cases during the 12th and 16th epi-weeks, respectively. On the 20th epi-week (August), a sudden increase was noticed to reach the highest in September and October. During the same period of the pandemic, a total of 982 TB cases were reported. An average of 177 TB cases per week were reported during the first 8 epi-weeks (1 to 8). From epi-week 9 till epi-week 16, only 158 TB cases were notified weekly. This was increased to reach 193 cases weekly within the last 12 epi-weeks (17 to 28). The notification was gradually decreased in August and September 2020 to reach 15% in October 2020.

**Figure 2.**
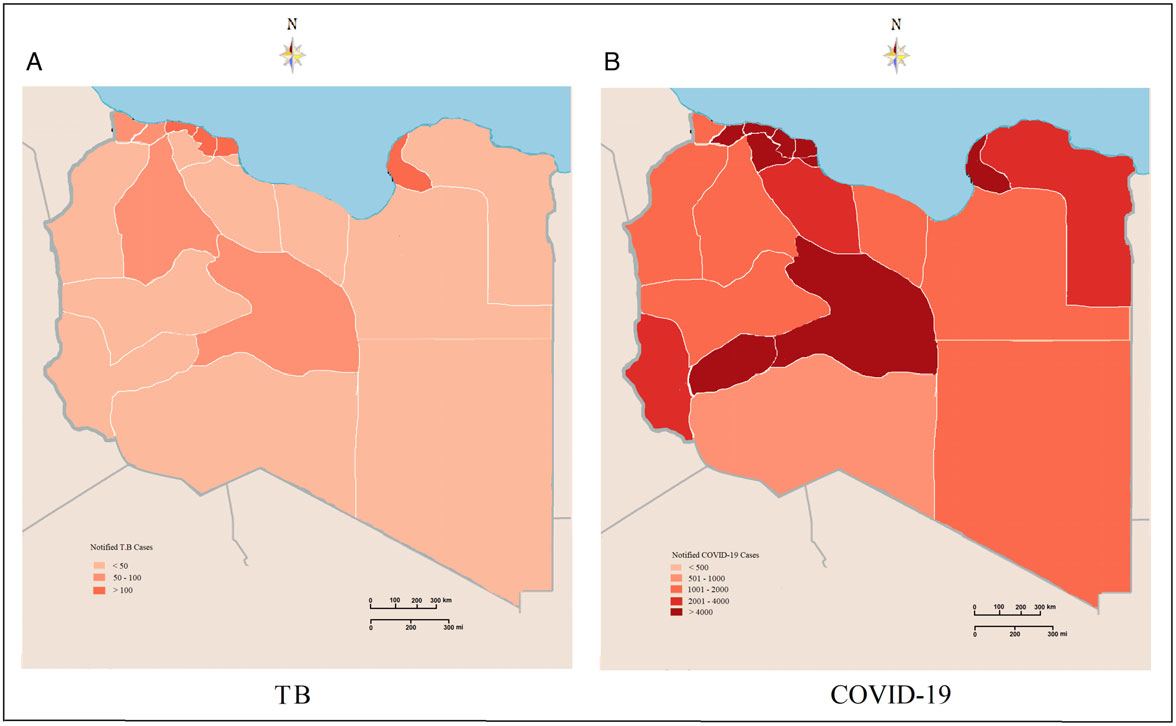
Spatial distribution of notified TB **(A)** and COVID-19 **(B)** cases during the pandemic period in Libya.

The spatiotemporal analysis of TB and COVID-19 was illustrated in Figure 2. Figure 2A shows the geographic and spatial distribution of TB in Libya during the pandemic period. There is a significant variation in the spread of TB through all the country. The overall prevalence was high mainly in the western and eastern regions, followed by southern and central regions. At the county level, the highest TB prevalence was reported in Tripoli, Benghazi, and Sebha. TB prevalence showed a significant low pattern in Saharan areas at the southern and eastern regions.

As of March 23, 2020, 22 districts (of all Libya) reported 41 686 COVID-19 cases with the number of incident cases ranging from 3 to 691 per day. The average incidence rate was 11.5 infections per 100 000 persons (range: 0.05–494) during the selected period of the COVID-19 pandemic. The geographic distribution and variation of COVID-19 cases are illustrated in Figure 2B. The spatial patterns of COVID-19 were dense all over the country. The western and southern regions consistently portrayed a higher prevalence of COVID-19 during the pandemic period (32 epi-weeks), followed by the eastern and western mountain regions. On the county levels, the highest prevalence was reported in Tripoli, Benghazi, and Sebha, from where the contagions follow the pandemic spread all over the country.

**Figure 3.**
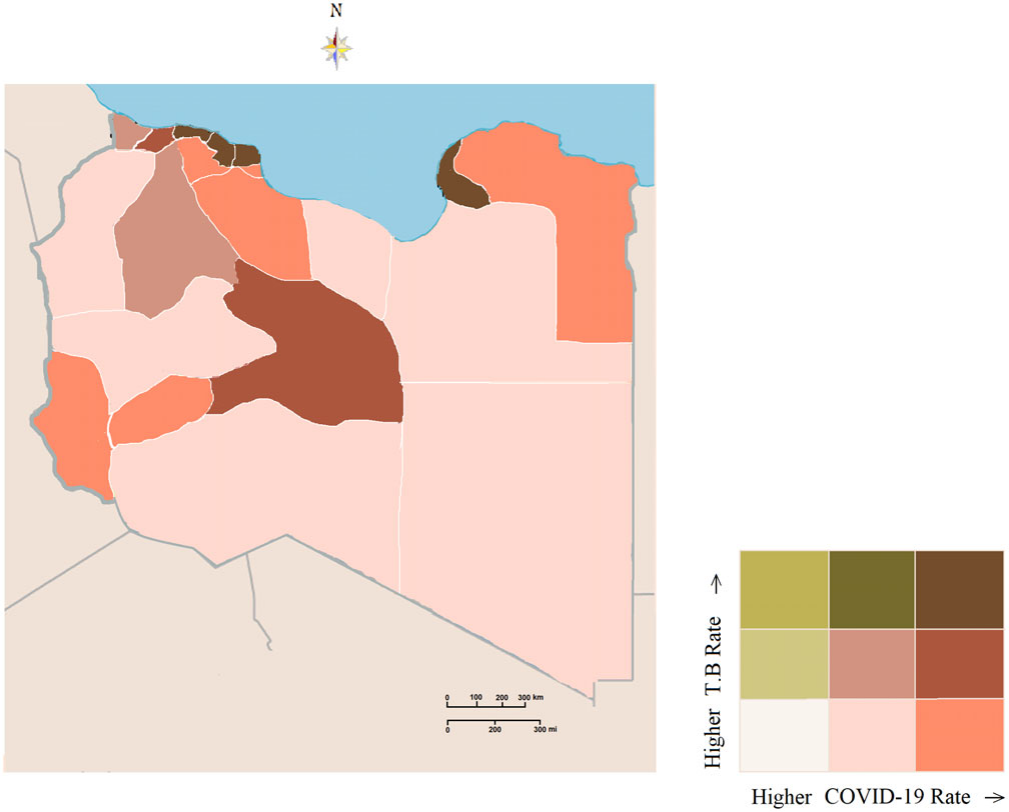
COVID-19 and TB rates in Libya. A color combination (high, medium, low) has been used to display the relationship between COVID-19 and TB.

To illustrate the spread of the 2 diseases and the geographic combination between them, we plotted the temporal changes in COVID-19 and TB during the pandemic period, as shown in Figure 3. The map portrays what appears to be high co-occurrences in certain locations of the study area. Tripoli, Sebha, and Benghazi consistently showed higher incorporation patterns of the 2 intertwined infections. Conversely, the western mountain region and southeast region of the study area exhibited a lower concordance. This shows a clear spatiotemporal geographic association between TB and COVID-19, which may indicate a synergistic impact.

In this study, we explored the patterns of spatiotemporal dynamics of TB and COVID-19 during the spread of the pandemic. Our data showed distinct spatiotemporal clustering patterns between both diseases, which could be used to implement new strategies to tackle the consequences of these diseases. However, further studies are needed to highlight the factors that contribute to the spread and mortality of the 2 epidemics. TB has a long and varying incubation period, and thus it is expected to increase in the post-pandemic era of COVID-19.^8^ Hence, concerns should be raised on the scale of the TB/COVID-19 pandemic in Libya and the uncertainty of preventive programs that disrupted not only the pandemic, but also the ongoing armed conflict.^8,9^
